# The Focal Play Therapy: An Empirical Study on the Parent–Therapist Alliance, Parent–Child Interactions and Parenting Stress in a Clinical Sample of Children and Their Parents

**DOI:** 10.3390/ijerph17228379

**Published:** 2020-11-12

**Authors:** Ilaria Chirico, Federica Andrei, Paola Salvatori, Irene Malaguti, Elena Trombini

**Affiliations:** Department of Psychology, University of Bologna, 40126 Bologna, Italy; federica.andrei2@unibo.it (F.A.); paola.salvatori2@unibo.it (P.S.); irene.malaguti@gmail.com (I.M.); elena.trombini@unibo.it (E.T.)

**Keywords:** psychodynamic child psychotherapy, child–parent psychotherapy, therapeutic alliance, parenting stress index (PSI), parent–child interactions, parenting, developmental psychopathology, infant mental health, child eating disorders, child evacuation disorders

## Abstract

The present study aims to investigate the outcomes of the Focal Play Therapy with Children and Parents (FPT-CP) in terms of parent–therapist alliance, parent–child interactions, and parenting stress. Thirty parental couples (*N* = 60; 30 mothers and 30 fathers) and their children presenting behavioral, evacuation and eating disorders took part to the study. Through a multi-method longitudinal approach, data were collected at two time points (first and seventh sessions) marking the first phase of the intervention specifically aimed to build the alliance with parents, a crucial variable for the remission of the child’s symptoms (and to the assessment of the child’s symptoms within family dynamics.) Therapeutic alliance was assessed by the Working Alliance Inventory by therapists and parents. Parent–child interactions and parenting stress were evaluated using the Emotional Availability Scales and the Parenting Stress Index, respectively. Results showed that a positive parent–therapist alliance was developed and maintained during the first seven sessions. Furthermore, parent–child interactions significantly improved on both parents’ and child’s dimensions. However, parenting stress levels remained unchanged between the two time points. The findings should enrich scientific knowledge about the role of parental engagement in preschool child-focused treatments as to better inform practice and improve the quality of care for children and their families.

## 1. Introduction

Early parent–child interactions have a major influence on child’s cognitive, emotional and social development [1,2,3]. Their bidirectional nature is well documented (i.e., parents influence children just as vice versa) [4,5]. Historically, the literature has emphasized various dimensions of adult–child interactions that affect child socio-emotional adjustment as well as the development of language and other cognitive abilities [6,7]. Among them, a growing body of evidence indicates that emotional availability (EA) represents a key determinant for positive parent–child interactions [8,9,10,11,12,13,14,15,16,17]. Based on attachment theory [18] integrated with Emde’s [19] perspective on emotions, EA denotes the quality of emotional exchanges between parents and their children with focus on their reciprocal accessibility and ability to read and respond appropriately to one another’s communications [20,21,22,23].

### 1.1. Parent–Child Interactions and Child Psychopathology

Empirical evidence has shown that problems in parent–child interactions are strictly associated with the development of child psychopathology [21,22,23,24,25]. In preschool years, child developmental tasks center around the acquisition of physical and emotional independence and autonomy [26]. In this context, parents need to balance protective and "letting go" behaviors to stimulate the development of the child’s self-regulatory abilities in different areas of his/her development [25,27,28,29,30,31,32,33,34,35,36,37,38,39]. The difficulties which may arise during this stage of development often lead to child behavioral, social and emotional problems, which become in turn common causes of concerns for parents of children aged 2–5 years [24,40]. Among them are child’s oppositional and aggressive behaviors, difficulty with eating and/or evacuation and so on. Particularly, eating disorders include: the avoidant/restrictive food intake disorder, eating of non-nutritive substances, repeated regurgitation and chewing of food, while evacuation disorders consist of constipation, enuresis and encopresis [41]. Without early interventions, these problems tend to persist into school age with negative consequences on the child’s physical and mental health and family burden [42,43]. Indeed, in most of these cases parents suffer from distress and psychological impairment [44,45,46,47].

### 1.2. The Focal Play Therapy with Children and Parents

In order to prevent adult psychopathology, most clinical approaches today focus on the early identification and treatment of problems in the parent–child relationships [48,49,50,51]. The Focal Play Therapy with Children and Parents (FPT-CP) [25,36,37,38,39,52,53] is a psychodynamic model of intervention originally developed for eating and evacuation disorders and then adapted to a wide range of problems usually connected to parent–child relationship problems during preschool years. It is based on both the active engagement of parents in the diagnostic-therapeutic process and the use of play as a narrative dimension of the family history [54,55,56,57].

The FPT-CP is structured into weekly alternate play sessions with children and parents together and sessions with parents only. Specifically, they are organized as follows: first session with parents; second session with the child and his/her parents; third session with the child and his/her mother; fourth session with parents; fifth session with the child and his/her father; sixth session with parents; seventh session with the child and his/her parents. Basically, during the FPT-CP joint sessions the therapist introduces the child to a temporal sequence of play where the main character is a plasticine puppet which performs the human basic physiological functions. It seems to enjoy eating and, afterwards, it expresses the need to go to the toilet in a potty made with plasticine. The focus is on the phenomenal qualities of both eating and evacuation of natural functions. In this context the therapist allows the child to project his/her psychological contents, desires, fears and internal conflicts into play. Parents are asked to take part in the play and, afterwards, they discuss with the therapist alone (without the child) what emerged during play sessions, the psychological meanings of the play and parental attitudes which may support (e.g., intrusive and coercive manners) or not support (e.g., tolerance, collaboration) the child’s symptoms. Positive parental skills are promoted along with the achievement of self-managed and self-regulated child behaviors into a harmonious family life [36]. 

Specifically, the FPT-CP first phase (seven sessions) is aimed to understand the child’s symptoms within family dynamics and to promote and maintain a positive therapeutic relationship with parents [25,36,37,38,39,52]. This aspect is crucial since the parent–therapist alliance allows parents to understand the child’s problems and to come to an agreement regarding the main goals and tasks of treatment [42,43,58,59,60,61,62,63,64].

Over the past few decades, a vast amount of literature has evolved around the topic of the alliance in individual psychotherapy, while similar research in family therapy, including the FPT-CP, is recent [65,66] and needs more empirical evidence. The few studies available on schoolchildren and their parents attending separate treatment sessions have shown that a positive parent–therapist alliance is associated with low drop-outs, a decreased youth symptomatology, and improved parenting practices and family functioning [67,68,69,70].

### 1.3. The Present Study

In light of the above-discussed issues, there is a need to collect data in order to better inform clinical practice and to improve the quality of care for children and their families. Specifically, through a multi-method longitudinal approach, the present study wants to investigate the outcomes of the FPT-CP first phase in terms of parent–therapist alliance, parenting stress, and parent–child interactions. Indeed, to our knowledge, while some evidence on the benefits of a positive alliance with parents—who are treated separately from their schoolchildren—exists, there is a paucity of data on the alliance with parents involved in the therapy sessions with their preschool children.

Data were collected at two time points (T1: first session, T2: seventh session) marking the first phase of the FPT-CP by recruiting a clinical sample of preschool children and their parents. In this context, we aimed to investigate differences between T1 and T2 in: (a) the parent–therapist alliance from each participant’s perspective; (b) the levels of parental distress; (c) the quality of mother–child and father–child interactions from both sides. We hypothesized that: (a) the parent–therapist alliance would be positive and stable over time; (b) parental distress would significantly decrease; (c) parent–child interactions would significantly improve.

## 2. Materials and Methods

### 2.1. Participants

Families were recruited consecutively between November 2015 and December 2017 at the "Psychological Consultation Centre for Children and Parents" (Department of Psychology, University of Bologna, Italy; director: Professor Elena Trombini). The center provides psychological assessment, treatment and support for children and their families. Parents were given voluntary access to the Centre for their child’s behavioral (e.g., oppositional and aggressive behaviors), eating (e.g., food refusal and selective eating), or evacuation (e.g., constipation, enuresis and encopresis) problems. 

This research adopted a longitudinal design. A total of 30 couples (*N* = 60; 30 mothers and 30 fathers) and their preschool children (*N* = 30; 21 males and 9 females) took part in the study. Exclusion criteria for the present study were: (a) child’s organic diseases, (b) child’s neurodevelopmental disorders, (c) parental past or present psychiatric disorders, (d) parents’ lack of competence in the Italian language, (e) the refusal of one parent to attend the study. No exclusion criterion was met by any of the families who participated in the study.

Seven psychotherapists carried out the FPT-CP with the children and their families. All therapists were female, experts in psychoanalytic psychotherapy with children and families, and had been previously trained to use the FPT-CP methodology

### 2.2. Procedure

The study was approved by the Ethic Committee of the University of Bologna (Italy). Participation was voluntary and based on the family informed written consent which included confidentiality and the client’s right to withdraw at any time. Each family was randomly assigned to a clinician according to his/her availability, and the average caseload for each psychotherapist was about four families, each of which was seen once a week. 

At the end of the first and sixth sessions parents were asked to complete a demographic questionnaire and two self-reports on therapeutic alliance and parenting stress, respectively. Data on alliance were triangulated with the scores obtained by the therapists at the same measure, which they filled in for both mothers and fathers at T1 and T2. Changes in the quality of the parent–child interactions were evaluated at the beginning of the second (before treatment) and during the seventh (where only data collection occurred) sessions. To this aim, two consecutive sessions of a 10-minutes free-play interaction, first with the mother and then with the father, were recorded.

### 2.3. Measures

The therapeutic alliance refers to the quality of the relationship between the client and the therapist. Specifically, it consists of three dimensions: the agreement on (1) goals of treatment; (2) tasks, methods and activities used to achieve treatment goals; (3) the development of a personal bond between the client and the therapist [71]. Therapeutic alliance was assessed by the *Working Alliance Inventory-Short Form* (WAI-SF) [72,73]. The WAI-SF is composed by 12 items rated on a seven-point Likert scale. The total score (range 12–84) is based on the sum of three subscales: goal, tasks, and bond (range 4–28). Higher scores indicate a more positive alliance [74].

Parental distress arises when the demands associated with parental role exceed parent’s resources to face them [75]. In the present study parental distress was measured using the *Parenting Stress Index—Short Form* (PSI-SF) [76,77]. The PSI-SF consists of 36 statements which evaluate specific domains of parental distress rated on a 5-point Likert scale. The total score (range 36–180) is a combined score of the three subscales (range 12–60): parental distress, parent–child dysfunctional interaction and difficult child. As indicated by the Italian validation [77], scores between the 15th and 84th percentiles are within the normal range for stress; scores between the 85th and 89th percentiles represent a high level of stress; scores ≥ 90th percentile indicate clinically significant or severe parenting stress. 

Interactions between parents and their children were coded through the fourth edition of the *Emotional Availability Scales: Infancy to Early Childhood Version* (EAS) [78]. The EAS have been largely used in research settings over 20 countries to evaluate the quality of parent–child relationships focusing on emotional availability which refers to the quality of emotional exchanges between parents and children [79]. These scales describe and evaluate six dimensions, four on the adult’s side (Sensitivity, Structuring, Nonintrusiveness, and Nonhostility), and two on the child’s side (Responsiveness to adult and Involvement of adult). Scores are assigned based on the frequency and the quality of the behavior observed. For each dimension, a total score and a direct score can be obtained. Direct scores for each dimension are evaluated on a 1–7 points Likert scale where the lowest scores (1–2–3) indicate severe clinical problems, the mid-point ratings (4–5) refer to mild/moderate problems, the high-end scores (5.5–6–7) represent good/optimal ratings. In the present study, direct scores were used, as common for research purposes, in order to give an immediate indication of the level of emotional availability displayed by the dyad [80,81]. All videos were scored by two blind raters previously trained in the use of the EAS. The degree of agreement between the two coders was measured using the average absolute agreement intraclass correlation coefficients (ICC) [82] on a random selection of 30% of the videos. ICCs averaged 80. 

### 2.4. Statistical Analysis

Demographic data were analyzed using Pearson’s χ^2^ test and Student’s *t* test for independent samples for nominal and continuous variables, respectively. All hypotheses were tested through Repeated measure Analyses of Variance (ANOVA) or Multivariate ANOVA (MANOVA). Each model included Role (i.e., mother vs. father and parent vs. therapist in the case of the analysis on therapeutic alliance), and Time (i.e., T1 vs. T2) as within-subject variables. All statistical analyses were performed using SPSS (version 25) for Windows (IBM, Armonk, NY, US). A *p* value of less than 0.05 was considered significant.

## 3. Results

### 3.1. Demographic and Clinical Characteristics

All couples were Italian, employed, married (83.3%) or cohabiting (13.3%). No significant differences between parents were found with regard to education level as both mothers (77%) and fathers (67%) had mostly a university degree. Differences in age reached significant levels, with fathers being slightly older than mothers (*t* = −2.53, *p* < 0.05; *M* = 39.9, *SD* = 4.9, and *M* = 42.1, *SD* = 5.1, for mothers and fathers, respectively). Children’s ages ranged between 2.1 and 5.8 years (*M* = 4.1, *SD* = 1.1), and they were referred for behavioral (43.3%), evacuation (36.7%) or eating (20%) problems.

### 3.2. Therapeutic Alliance 

Table 1 presents statistics for the WAI-SF total scores at T1 and T2. Parents and therapist’s alliance scores were high and indicative of a positive alliance at each time of assessment. Results from the ANOVAs showed that showed no significant main effects of Role, Time, nor their interaction over WAI global scores (all *p*s > 0.05). With regards to the ANOVA pertaining therapist’s ratings, a main effect of Role was detected, as alliance with mothers was overall higher than alliance with fathers (*F* (1, 29) = 12.8, *p* < 0.001). Moreover, therapist’s ratings on alliance were significantly lower compared to self-rated alliance by both mothers (*F* (1, 29) = 32.1, *p* < 0.001) and fathers (*F* (1, 29) = 26.3, *p* < 0.001). No significant main effect of Time nor interaction effect was detected (all *p*s > 0.05).

### 3.3. Parental Distress

Table 2 presents descriptive statistics for the PSI scales and global scores at T1 and T2. ANOVAs’ results showed no significant difference in parenting stress levels between fathers and mothers (all *p*s > 0.05). These findings seem to attest that parenting stress remained essentially unchanged from session 1 to session 6. In light of these results, PSI’s scores obtained at T1 and T2 were averaged in order to obtain a more precise global indicator of parenting distress. This new score was used to check for the percentage of parents above the cut-off points suggested by the PSI manual. In the present sample, 11 mothers (36.7%) and 7 fathers (23.3%) reported clinically relevant distress levels, 3 mothers (10%) and 4 fathers (13.3%) were considered at risk, and 16 mothers (53.3%) and 19 fathers (63.3%) scored below the threshold level. 

### 3.4. Parent–Child Interactions

Table 3 presents the results of two repeated measure MANOVAs, comparing separately parent’s and child’s dimensions of the EAS. With regards to parent’s dimensions of the EAS, results showed a significant main effect of Role, Time and EAS dimensions, as well as significant interaction effect of Time × EAS dimensions (all *p*s < 0.05). Particularly, scores at the EAS were overall significantly higher for mothers compared to fathers. Significantly different mean values emerged across dimensions for both parents, with Non-hostility being the dimension with the highest scores and Structuring the dimension with the lowest (*p* < 0.001). Moreover, with the exception of Non-hostility which remained almost constant, each dimension improved significantly from T1 to T2 irrespective of parental role (see Table 3). This result may indicate a positive effect of the FPT-CP over parents’ interactions with their children. Despite such improvement, when the Structuring dimension was considered, mothers (T1: *M* = 4.6, T2: *M* = 4.9) and fathers (T1: *M* = 3.8, T2: *M* = 4.6) reached scores below the cutoff point for an optimal interaction at both assessments. Yet, at T2, fathers’ scores on the Sensitivity dimension were still not optimal (T1: *M* = 4.6, T2: *M* = 5.1).

With regard to the child dimensions of the EAS, MANOVA’s results showed significant main effects for all the variables included in the model; namely, Time, Role and EAS dimensions (all *p*s < 0.05), while no interaction effect emerged (*p*s > 0.05; see Table 3). Along the line of parent’s dimensions, scores at the child’s dimensions were higher for mothers compared to fathers. Similarly, scores improved significantly from T1 to T2 for both Responsiveness and Involvement, thus suggesting a positive effect of the FPT-CP over children’s interactions with their parents. Nevertheless, with regard to child–father interactions, children’s scores on Sensitivity were still not optimal at T2 (T1: *M* = 4.4, T2: *M* = 5.1), while scores at Involvement fell below the critical threshold level (T1: *M* = 3.8, T2: *M* = 4.4). 

MANOVAs were rerun by controlling separately for the effects of Child’s Age (used as a continuous covariate), Child’s Diagnosis (i.e., behavioral, evacuation, eating problems), and Parental Distress (i.e., above vs. below the clinically relevant threshold of the 85th percentile), as relevant variables which might have altered the results. Each model included one of these variables together with Role and Time, and was run twice, once for parent’s and once for child’s dimensions of the EAS. Child’s Age and Diagnosis did not contribute significantly to explain the differences in parent–child interactions (all *p*s = n.s.); main effects of Time, Role and EAS dimensions remained significant. With regards to Parental Distress, no new effects were detected for parent’s dimensions of the EAS (all *p*s = n.s.). When child’s dimensions were considered, an additional significant interaction Parental Role × Parental Distress was found (*F*(1, 26) = 7.25, *p* < 0.05). This result indicates that, during the interaction, children present lower levels of both Responsiveness (T1: *M* = 3.9, T2: *M* = 4.4) and Involvement (T1: *M* = 3.1, T2: *M* = 3.6) if their fathers scored above the clinically significant threshold on parenting distress, compared to children whose fathers show normal distress levels (Responsiveness: T1: *M* = 4.8, T2: *M* = 5.5; Involvement: T1: *M* = 4.2, T2: *M* = 4.8). On the other hand, children’s interaction with mothers was not affected by their stress levels (i.e., above vs. below the clinically relevant threshold of the 85th percentile). 

## 4. Discussion

The goal of this study was to investigate the outcomes of the FPT-CP in terms of parent–therapist alliance, parental distress, and parent–child interactions in a clinical sample of preschool children. Our findings support the hypotheses concerning parent–therapist alliance and parent–child interactions except for parental distress which did not significantly decrease at T2. The FPT-CP is a clinical methodology based on the need for the therapist to build an early, positive, therapeutic relationship with children and their parents in order to facilitate the treatment success. As previously described, parents are actively involved in play sessions with children and afterwards in sessions with the therapist alone where they share comments and reflect on the psychological meanings of the child’s and family’s play. The recent international literature in this field has shown that a positive therapeutic relationship with parents significantly correlates with low premature terminations from therapy, increased youth functioning and family well-being across different types of child and family treatment [65,66,67,68,69,70]. However, today, there is a dearth of data on preschool child-focused interventions where parents are actively involved with the aim of understanding the child’s symptoms within family dynamics and restoring healthy family relationships.

In line with our expectations, results have shown that a positive parent–therapist alliance was developed and maintained during the first FPT-CP 7 sessions. In this regard, it is important to consider that parents’ access to the Centre was voluntary, subsequently their treatment motivation was presumably high, and they were also well-educated and has been willing to share aims and procedures of the intervention since their first sessions. In line with previous studies [83,84], we did not find differences between mothers’ and fathers’ alliance scores, which were significantly higher than the therapist’s ratings of alliance. However, unexpectedly, the therapist’s report of the alliance with mothers was significantly higher than the alliance with fathers at both measurements. Although the therapists in this sample were all females and more gender similarities might explain these results, several studies did not find significant relationships between therapist alliance and treatment outcomes. Thus, the ability of therapists to accurately evaluate various aspects of their treatments was questioned [85,86,87].

For what concerns levels of parenting stress, significant differences did not emerge neither between T1 and T2 nor between mothers and fathers. Nevertheless, at a qualitative level, mothers and fathers showed a different pattern of stress development on the Difficult Child scale. This dimension deals with how parents perceive their children, whether they are easy or difficult to care for [76,77] and this is often used in studies with clinical samples of children whose parents struggle to manage their behaviors. While at the end of the FPT-CP first phase, mothers’ scores were lower and in a subclinical range (T1: 85th percentile, T2: 80th percentile), fathers’ ratings slightly increased and still had clinical significance (85th percentile). It may be that, during the FPT-CP 7 sessions, mothers started to understand the reasons behind their children’s maladaptive behaviors and symptoms rather than to simply perceive them as difficult, challenging or disturbing. A similar result did not occur in the sample of fathers who probably needed a longer therapeutic process to fully understand the psychological meanings behind the child’s behaviors and to be able to effectively deal with them. Indeed, although they are highly motivated and psychologically activated, they can perceive therapeutic tasks as difficult as they are not yet equipped with the emotional skills necessary to get through them.

In line with our hypothesis, with regard to parent’s dimensions of the EAS, both mother–child and father–child interactions significantly improved from T1 to T2 except for the scale of Non-hostility on which parents obtained the highest scores among the EAS dimensions at both time points. Indeed, there was no evidence of parents’ negative emotionality towards their children both in its covert and overt components. However, although significant improvements occurred in parent–child interactions, at the end of the FPT-CP first phase fathers’ scores on the Sensitivity scale were not yet optimal suggesting, according to the manual [78], the presence of some inconsistencies of parental behaviors, the lack of a proper sense of timing and some difficulties with dealing with conflict situations. Furthermore, both parents still reported problems on the Structuring scale which measures the caregiver’s ability to scaffold the child’s activities and set appropriate limits while respecting the child’s need of autonomy. As previously discussed, in the preschool years children strive for independence and autonomy and, at this stage, parents should effectively adapt themselves to understand children’s desire to do things for themselves and support their emerging autonomy, thus preventing child behavioral, social and emotional problems in such a delicate developmental phase. Due to the nature of problems in our sample, this aspect of parent–child relationships may require more sessions to effectively reach optimal levels of interaction. Furthermore, mothers’ scores on the EAS scales were overall significantly higher than fathers at both time points, thus suggesting a higher level of maternal relational competence which has been present since the beginning of the intervention.

For what concerns child’s dimensions of the EAS, as expected, they significantly improved with mothers and fathers. Furthermore, at both measurements, child’s scores were higher for mothers compared to fathers, thus confirming a less problematic scenario in the context of child-mother relationships. Indeed, as for child-father interactions, at T2 children’s scores on the Responsiveness scale (i.e., the counterpart of the adult Sensitivity scale) were not still optimal. Specifically, this dimension measures both the child’s responsiveness to the adult and the presence of autonomous activities and explorations [78]. In this sample, children showed an affectively positive and responsive attitude towards their fathers although responsive and exploratory behaviors were somehow unbalanced in favor of the former. Moreover, moderate problems were reported on the Involvement scale as children showed some over-involving behaviors towards their fathers that, according to the EAS manual [78], might suggest that they were assuming the lion’s share of the responsibility for maintaining contact and interactions with the adult, thus compromising the child’s autonomous initiatives.

The overall differences found on the EAS scales for both parent’s and child’s dimensions were not explained by neither child’s age nor child’s problems. However, while children’s interactions with mothers were not affected by their stress levels, it was found that children obtained lower scores on the Responsiveness and Involvement scales when fathers’ ratings of distress were clinically significant compared to children whose fathers showed normal distress levels. It seems that, compared to fathers, maternal distress did not hamper the capacity of the mother–child dyad to share an emotional connection and to enjoy a mutually fulfilling and healthy relationship. We can speculate on a greater maternal ability to manage stress in ways the children found reasonable.

Overall, the results show that a positive therapeutic relationship with both parents was developed and maintained during the first seven sessions as necessary condition for the success of the FPT-CP. Along with it, significant changes in parent–child interactions occurred for mothers, fathers and their children toward more positive healthy relationships. These results are clinically relevant and give advice about the importance of involving parents in child-focused treatments where a structured clinical methodology is used. In the present sample mothers showed somehow a less problematic scenario and a greater parental competence since the beginning of the intervention. Hence, despite significant paternal improvements, mothers and fathers may follow a different trajectory for individual changes and fathers might need more therapeutic sessions to build and maintain a healthy relationship with their children. In this regard, clinicians should carefully monitor progresses as well as mothers’ and fathers’ individual times to achieve them. More studies are needed on this issue with longer research-design.

The following limitations have to be considered. At first, a larger sample size is required for more reliable results with greater precision and power. Indeed, children’s limited sample size could not have allowed to detect specific differences related to child’s diagnosis (behavioral, evacuation, eating disorders). Furthermore, since parents’ access to the Centre was voluntary and all parents were well-educated a sampling bias could have occurred. Similarly, using a more balanced sample of therapists (not women only) may allow to better understand some discrepancies found among the therapists’ and parents’ scores. To this aim, in order to obtain more reliable data on therapeutic alliance, the use of both questionnaires and observational measures such as the System for Observing Family Therapy Alliances (SOFTA) [88] would be useful. Moreover, the present study mostly focused on the adults’ voice, while data on parent–child interactions were collected from the child’s side as well. In order to obtain a more comprehensive picture of adult and child-related data [89,90,91,92] future studies should capture children’s voices in reliable and valid ways. It would also be interesting to collect data longitudinally over the course of the FPT-CP to monitor clinical outcomes in terms of both parental variables and remission of child’s symptoms. Lastly, we could not infer causality due to the nature of the study and, therefore, future research should compare different treatment approaches based on different levels of parental engagement as to understand how these effectively work, thus improving the quality of care for children and their families.

## 5. Conclusions

The FPT-CP is a structured psychodynamic methodology aimed to promote and maintain the parent–therapist alliance and it is based on the use of play as a narrative dimension of family dynamics. Parents are actively involved into the child’s play sessions where they gradually understand the psychological meanings behind child’s play, his/her resources and capabilities well beyond child’s symptoms. In this way, parents become aware of the role they play in regard to child’s difficulties and the child is no longer seen as simply difficult or challenging. As a consequence, parents are motivated to change parental non-adaptive behaviors to help their children and family dynamics can improve in a more compatible way with the child’s developmental needs.

In the present study, we offered empirical evidence on the associations between the use of the FPT-CP and the improvement in parent–child interactions since its first sessions. Therefore, in light of what previously discussed and of the empirical evidence collected in several studies, this methodology could represent an innovative model for preventive psychodynamic interventions to be applied to both public and private clinical contexts for children and their families.

## Figures and Tables

**Table 1:** Means ± Standard Deviations of the WAI-SF global scores for mothers, fathers and therapists, at T1 and T2.

Role in the Session	Mothers	Fathers	Therapists	
			Mothers	Fathers
Time of assessment				
T1	71.3 ± 7.8	68.7± 10.6	61.9 ± 8.5	58.5 ± 10.7
T2	71.2 ± 8.5	68.6 ± 8.9	63.1 ± 10.2	59.3 ± 10.8

Note. WAI-SF = Working Alliance Inventory—Short Form. T1 and T2 refer to session number 1 and 6, respectively.

**Table 2:** Means ± Standard Deviations of the PSI dimensions and global score for mothers and fathers at T1 and T2.

	Mothers		Fathers	
	T1	T2	T1	T2
PSI dimensions				
Parental distress	28.8 ± 9.2	29.2 ± 8.1	27.5 ± 6.8	27.4 ± 7.2
Parent–child dysfunctional interaction	24 ± 6.8	23.3 ± 6.8	22.1 ± 6.1	23.1 ± 6.8
Difficult child	32.3 ± 8	31.1 ± 7.1	30.7 ± 5.7	31.4 ± 7.1
Defensive responding	18.3 ± 6.1	18.1 ± 5.5	16.7 ± 4.4	16.6 ± 4.5
Global score	85.1 ± 19.9	83.7 ± 18.2	80.3 ± 12.9	81.9 ± 16.3

Note. PSI = Parenting Stress Index. T1 and T2 refer to session number 1 and 6, respectively.

**Table 3:** Comparisons between mothers and fathers at the two assessments of the parent’s and child’s dimensions of the EAS.

	Mothers		Fathers		Time	Parental Role	EAS Dimensions	Time * Parental Role	Time * EAS Dimensions	Parental Role * EAS Dimension
	T1	T2	T1	T2						
EAS - parent					*F* = 9.5, *p* < 0.01	*F* = 5.1, *p* < 0.05	*F* = 60.1, *p* < 0.001	*F* = 1.9, *p* = n.s.	*F* = 7.2, *p* < 0.01	*F* = 2.4, *p* = n.s.
Sensitivity	5.3 ± 1.2	5.8 ± 1.1	4.6 ± 1.3	5.1 ± 1.3						
Structuring	4.6 ± 1.2	4.9 ± 1.1	3.8 ± 1.3	4.6 ± 1.5						
Non-intrusiveness	5.6 ± 1.2	5.9 ± 1.2	5.5 ± 0.9	5.9 ± 0.8						
Non-hostility	6.5 ± 0.7	6.5 ± 0.6	6.3 ± 0.7	6.4 ± 0.8						
EAS - child					*F* = 12.2, *p* < 0.01	*F* = 7.2, *p* < 0.05	*F* = 24.7, *p* < 0.001	*F* = 0.19, *p* = n.s.	*F* = 0.1, *p* = n.s.	*F* = 1.3, *p* = n.s.
Responsiveness	4.9 ± 1.4	5.5 ± 1.3	4.4 ± 1.3	5.1 ± 1.4						
Involvement	4.4 ± 1.2	5.3 ± 1.3	3.8 ± 1.4	4.4 ± 1.6						

Note. EAS = Emotional Awareness Scales. T1 and T2 refer to session number 2 and 7, respectively. * refers to interaction effects.
